# Impact of delayed removal of pectus bar on outcomes following Nuss repair: a retrospective analysis

**DOI:** 10.1186/s13019-024-02685-z

**Published:** 2024-03-28

**Authors:** Der-En Keong, I-Shiang Tzeng, Nay Htut, Yu-Jiun Fan, Min-Shiau Hsieh, Yeung-Leung Cheng

**Affiliations:** 1 Division of Thoracic Surgery, Department of Surgery, Taipei Tzu Chi Hospital, Buddhist Tzu Chi Medical Foundation, New Taipei City, Taiwan; 2 School of Medicine, Tzu Chi University, Hualien, Taiwan

**Keywords:** Pectus excavatum, Nuss procedure, Nuss bar removal, Complications, Outcomes

## Abstract

**Background:**

Usually, pectus bars are removed 3 years after the Nuss procedure in patients with pectus excavatum. However, the optimal timing for postoperative pectus bar removal remains undefined. Our study investigated the effects of delayed pectus bar removal after Nuss repairs.

**Methods:**

Retrospective data were collected on patients who underwent Nuss procedures for pectus excavatum and had their bars removed from August 2014 to December 2020. Patients with correction periods > 3 years were divided into group A (< 6 years) and group B (≥ 6 years). Propensity score matching was used to compare complications and radiological outcomes associated with bar removal.

**Results:**

Of the 542 patients who underwent bar removal, 451 (Group A: 419 patients, Group B: 32) had correction duration > 3 years. The average correction duration was 4.5 ± 1.4 years. After propensity score matching analysis, group B [median duration: 8.0 (6.0–16.2) years] exhibited significantly longer median operative times (85 vs. 55 min; *P* = 0.026), higher callus formation rates (68.8% vs. 46.9%; *P* = 0.029), and greater median intraoperative blood loss (35 vs. 10 mL; *P* = 0.017) than group A [median duration: 4.2 (3.0–5.9) years]. However, following bar removal, the groups showed no statistical differences in the surgical complication rates (group A: 6.3% vs. group B: 9.4%; *P* = 0.648) or median ratio of radiological improvement (an improvement on the Haller index on chest radiography; 21.0% vs. 22.2%; *P* = 0.308).

**Conclusions:**

Delaying pectus bar removal after Nuss repair presents certain challenges but does not compromise overall outcomes. These findings suggest that a longer correction period may be unnecessary. However, further multicenter studies with long-term follow-up are warranted to assess long-term outcomes.

## Background

Pectus excavatum (PE), a congenital condition causing chest wall depression, can cause discomfort and reduced physical tolerance [1–4]. The Nuss procedure, a minimally invasive procedure introduced by Dr. Donald Nuss in 1998, corrects PE using metal bars [5–12], which are removed after 2–3 years. This technique is preferred over the Ravitch method and enjoys widespread use [13]. Early bar(s) removal risks incomplete correction, while longer retention periods may be warranted because of patient preferences or medical conditions [14, 15]. We examined the effects of delayed bar removal on complications and outcomes to provide insights into extended correction benefits and challenges of bar retention beyond the standard timeframe.

## Methods

### Participants

**Fig. 1:**
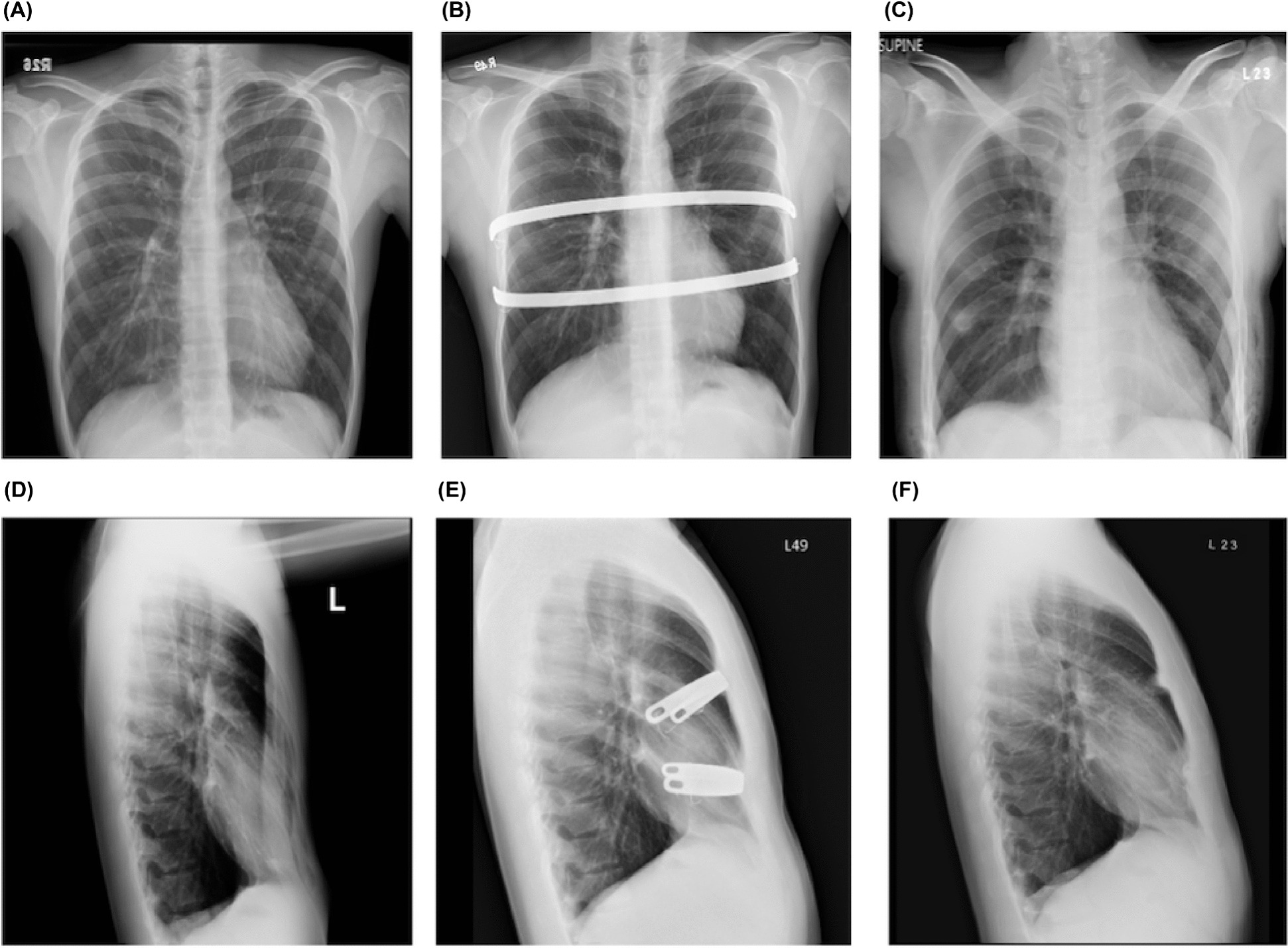
Haller index measurement.  Haller index measurement using posteroanterior and lateral chest radiographs before Nuss repair ( A , D ; CXR-HI: 4.32), before ( B , E ; CXR-HI: 2.35) and after ( C , F : CXR-HI: 2.35) bar removal 4 years post-surgical correction. CXR-HI, Haller index on chest radiography

The study was approved by the Ethics Committee and Institutional Review Board (IRB) of Taipei Tzu-Chi Hospital (Taipei City, Taiwan, Republic of China) (IRB No: 11-XD-109). Due to the study’s retrospective nature, the IRB exempted the need to acquire patient consent. The study included patients with PE who underwent a Nuss procedure and subsequent removal of the pectus bar in our institution between August 2014 and December 2020. Patient information was collected from hospital records, which included patients’ body mass index (BMI); age at the time of repair and removal; preoperative Haller index (HI), measured using pre-Nuss repair chest computed tomography (CT-HI); HI on chest radiography (CXR-HI) before repair and removal, after removal (Fig. 1), and during follow-up; operating times; the amount of blood loss; the presence of callus formation around the bars; duration of hospital stays; and any reported complications. The following exclusion criteria were applied: cases where the bar was removed within 3 years after repair, cases of recurrence after traditional surgery following the Nuss procedure, and cases with incomplete follow-up data. Patients who underwent correction for > 3 years were categorized into two groups: group A (< 6 years) and group B (≥ 6 years).

### Surgical techniques for bar removal

**Fig. 2:**
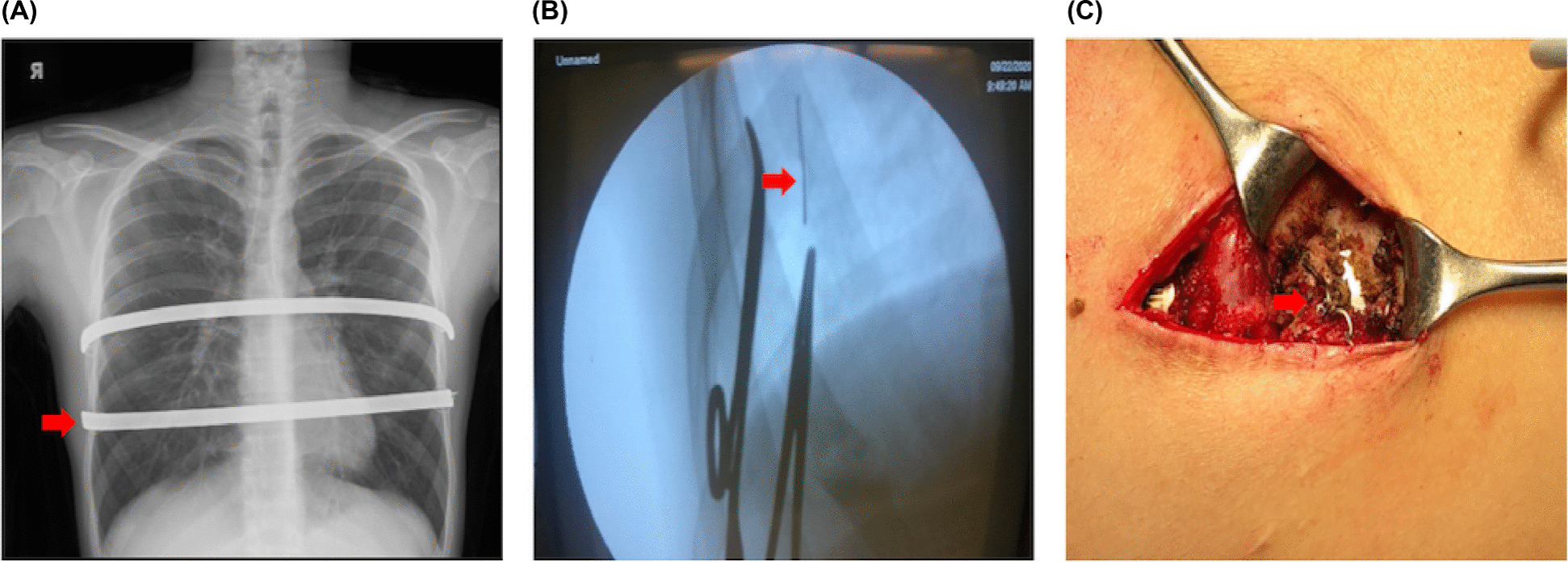
Radiologic and perioperative features.  Radiologic and perioperative features with bar removal 5 years post-correction. A  Segmental wire fracture on the right of the lower pectus bar (red arrow). B  Wire fragment (red arrow). C  Callus formation encasing wire (red arrow). CXR, chest radiograph

The surgical technique primarily used was previously described [11, 16]. Patients were placed in the supine position following the administration of anesthesia via a solitary-lumen endotracheal tube. The incisions for bar removal were made through the existing surgical scars. Subsequently, the surgeon proceeded to dissect the subcutaneous layers, uncovering both ends of the bars and previous steel wire fixation compounds [11, 16]. Any broken wire fragments identified on the preoperative chest radiograph (Fig. 2A) were subsequently removed using either palpation or C-arm fluoroscopic guidance (Fig. 2B). If a bony callus was found to cover the ends of the bar (Fig. 2C), it was meticulously removed using a rongeur to fully uncover the ends of the bar. After exposing the bar ends, the pectus removal bender (Zimmer Biomet, Jacksonville, FL, USA, or CHENTIAN, Taiwan) was utilized to partially straighten the right end of the bar, facilitating its withdrawal through the left side without rotation. Initially, sternal erosion assessment involved chest radiographs and the application of a nylon tape, which remained post-removal for intraoperative bleeding evaluation. Presently, we rely solely on chest radiographs without additional techniques. To ensure safety against potential bleeding, our protocol involves consulting Cardiovascular surgeons and Anesthesiologists, conducting preoperative conferences for high-risk procedures, and ensuring standby Cardiovascular teams are present, especially during bar removal in cases prone to significant bleeding. To manage local oozing effectively, absorbable hemostatic gauze was applied. The wounds were closed using the standard layered fashion without the use of drainage. Lastly, a 6-inch elastic bandage was expertly secured around the chest, delivering controlled compression to the surgical areas and fostering favorable healing conditions.

### Postoperative care and follow-up

Chest radiography (CXR) was conducted after the removal of the bar(s). To address postoperative pain, a regimen involving intravenous nonsteroidal anti-inflammatory drugs was administered. Patients who maintained hemodynamic stability were generally discharged on the day after the surgery. Follow-up appointments with the chest surgeon in the outpatient department were recommended at two weeks, six months, and annually after bar removal.

### Propensity score matching

Propensity score matching (PSM) was performed to balance the two groups regarding age, sex, BMI, and HI, as well as other known confounders, and improve comparability. PSM used a 1:1 nearest-neighbor approach for patients and was conducted using R software version 4.2.2.

### Statistical analyses

The Kolmogorov–Smirnov test was used to assess the distribution normality of the investigated parameters, with blood loss being the only one with a non-normal distribution. Continuous data were presented as median (range), while categorical data were reported as absolute values (%) by group. Differences between the two groups were compared using a two-sample t-test for continuous data and the Pearson chi-squared test for categorical data. Differences between the two groups were compared using the Mann–Whitney U test. All statistical assessments were two-tailed and considered significant when *P* < 0.05. Statistical analyses were performed using SPSS^®^ version 22 (SPSS Inc., IMB, USA) software.

## Results

Table 1 provides the clinical and demographic information of the 542 patients who underwent bar removal following treatment for PE using the Nuss procedure. The median age at repair was 23 years (range: 7–56), and the median age at bar removal was 27 years (range: 11–60). Male patients constituted 89.9% (487 individuals) of the cohort. The median body mass index (BMI) was 19.8 kg/m² (range: 13.8–28.6), indicating a varied distribution across the cohort. The HI had a median value of 3.91 before the operation (range 2.52–25.31). Regarding the history of pectus repair before removal, most patients had a primary Nuss repair (93.5%), some had a revision of the Nuss repair (5%), and very few underwent a Ravitch repair (1.5%). Most patients had two bars (73.2%, 397 patients), and a small number had three bars (5%, 27 patients). The median duration of correction was 4.1 years (range: 0.1–16.2 years). No blood transfusions were necessary. Complications were observed in a small subset of patients (4.8%, 26 patients), which were classified according to the Clavien-Dindo classification (CDC). The range of complications varied from mild occurrences, such as seromas or hematomas, pneumothoraxes, and wound dehiscence (requiring either observation or local treatments), to more severe complications, such as wound infections and prolonged chest pain requiring medication. No severe complications (CDC III/IV) were reported.

**Table 1:** Characteristics of the 542 patients with pectus excavatum who underwent bar removal after the Nuss procedure

Characteristics	Total (*n* = 542)	Group A (*n* = 419)	Group B (*n* = 32)
Age at repair, year, median (range)	23 (7–56)	23 (7–56)	25 (15–39)
Age at bar removal, median (range)	27 (11–60)	27 (10–60)	33 (21–47)
Sex, male, n (%)	487 (89.9)	412 (98.3)	30 (93.8)
BMI, median, (range kg/m2)	19.8 (13.8–28.6)	19.6 (13.8–28.6)	20.9 (15.61–28.54)
Haller index (preoperative), median (range)	3.91 (2.52–25.31)	3.83 (2.52–25.31)	4.19 (2.52–6.66)
History of pectus repair before removal, n (%)			
Primary Nuss repair	507 (93.5)	492 (100)	32 (100)
Previous Ravitch repair	8 (1.5)		
Revision of Nuss repair	27 (5.0)		
Number of bars, n (%)			
1	118 (21.8)	91 (21.7)	8 (25.0)
2	397 (73.2)	302 (72.1)	24 (75.0)
3	27 (5.0)	26 (6.2)	0 (0)
Duration of correction, years, median (range)	4.1 (0.1–16.2)	4.2 (3.0–5.9)	7.7 (6.0–16.2)
Operation time, min, median (range)	65 (35–210)	65 (40–140)	85 (45–210)
Estimate blood loss, mL, median (range)	10 (5–120)	10 (10–80)	35 (10–120)
Blood transfusion, n (%)	0 (0)	0 (0)	0 (0)
Hospital stays, days, median (range)	2 [2,–5]	2 ( 2–3)	2 (2,–5)
Complications, n (%)	26 (4.8)	23 (5.5)	3 (9.4)
Severity/type			
CDC I	17 (3.1)		
Seroma/hematoma: observation	4 (0.7)		
Pneumothorax: residual; observation	10 (1.8)		
Wound dehiscence: wound care	3 (0.6)		
CDC II	9 (1.7)		
Seroma/hematoma: local treatment	1 (0.2)		
Pneumothorax: catheter drainage	1 (0.2)		
Wound dehiscence: local treatment	1 (0.2)		
Wound infection: topical antibiotics	3 (0.6)		
Prolonged chest pain: medication	2 (0.4)		
CDC III/IV	0 (0)		

*BMI* body mass index, *CDC* Clavien–Dindo classification

Group A: <6 years of correction; group B: ≥6 years of correction

Furthermore, 451 patients had a correction duration of > 3 years and had complete preoperative and postoperative data for analysis. The average duration of correction was 4.5 ± 1.4 years. In Group A (*n* = 419), the median age at repair was 23 years (range: 7–56), aligning with a median age at bar removal of 27 years (range: 10–60). Males comprised 98.3% of Group A. The median BMI was 19.6 kg/m² (range: 13.8–28.6), and the median HI was 3.83 (range: 2.52–25.31). Among them, 91 (21.7%) had one bar, 302 (72.1%) had two bars, and 26 (6.2%) had three bars. The median duration of correction was 4.2 years (range: 3.0–5.9). Complications were reported in 5.5% of cases in Group A, graded by the CDC. In Group B (*n* = 32), the median age at repair and bar removal was 25 years (range: 15–39) and 33 years (range: 21–47), respectively. Male patients accounted for 93.8%. The median BMI in this group was 20.9 kg/m² (range: 15.61–28.54), with a median HI of 4.19 (range: 2.52–6.66). Regarding the number of bars, 8 (25.0%) had one bar and 26 (75.0%) had two bars. The median duration of correction was notably longer at 7.7 years (range: 6.0–16.2).

PSM yielded a population of 64 patients (32 in each of the two groups). Age, sex (male), BMI, and HI-CT before repair showed no significant (*P* = 0.214) differences between groups A and B after PSM (Table 2). After PSM, a subgroup analysis was performed comparing patients with a correction duration of 3–6 years (group A) and those with a correction duration of ≥ 6 years (group B). Significant differences between the two groups were noted in the age at bar removal (*P* < 0.001), duration of correction (*P* < 0.001), callus formation (*P* = 0.029), operative time (*P* = 0.026), and blood loss (*P*= 0.017). Nonetheless, no substantial disparities were observed concerning sex, BMI, HI, bar number, duration of hospitalization, complications, or radiographic enhancement [17]. The results of the analysis indicate notable variations in several demographic and clinical characteristics before and after PSM.

**Table 2:** Patient characteristics after PSM categorized by the time of correction

Variables	After PSM		
	A (*n* = 32)	B (*n* = 32)	* P *
Age at Nuss repair, year, median	24	25	0.141
Age at removal bar, year, median	28	33	< 0.001
Sex, male, n (%)	30 (93.8)	30 (93.8)	NA
BMI, median	20.4	20.9	0.652
HI (CT) before repair, median	4.08	4.19	0.214
Bar number, 2 or 3 (%)	26 (81.2)	24 (75.0)	0.123
Duration of correction years, median (range)	4.2 (3.0–5.9)	8.0 (6.0–16.2)	< 0.001
Callus formation, n (%)	15 (46.9)	22 (68.8)	0.029
Operation time, min, median (range)	55 (40–100)	85 (45–210)	0.026
Blood loss, mL, median (range)	10 (10–50)	35 (10–120)	0.017
Hospital stay, days, median (range)	2 (2–3)	2 [2–5]	0.349
Complications	2 (6.3)	3 (9.4)	0.648
HI (CXR) before repair, median	3.83	3.95	0.408
HI (CXR) before removal, median	2.85	2.92	0.398
HI (CXR) after removal, median	2.98	3.12	0.308
Improvement after repair (%), median	21.0	22.2	0.132

*BMI* body mass index, *PSM* propensity score matching, *HI* Haller index, *CT* computed tomography, *CXR* chest radiographs

Group A: <6 years of correction; group B: ≥6 years of correction

## Discussion

### Key findings

In our study, we found that delayed pectus bar removal following Nuss repair did not compromise overall patient outcomes. Notably, we observed no cases of massive bleeding during or after bar removal, which contrasts with findings from previous studies [18]. Additionally, our results suggest that longer correction periods may not be necessary.

### Comparison with similar research

In recent decades, the Nuss procedure has achieved broad acceptance as a surgical intervention for patients diagnosed with PE. The Nuss procedure has been consistently reported to exhibit a significantly shorter operation duration than the Ravitch procedure [19]. Regardless of the patient’s age, the Nuss procedure yields satisfactory cosmetic results. Although a higher recurrence rate of the deformity has been observed in younger individuals, the differences did not reach statistical significance (*P >*0.05) [6]. Previous studies have discussed the technique and its complications [20, 21]. and reported the optimal duration of correction as 2–3 years. However, in some cases, the bar may need to be removed earlier or later than the standard timeframe owing to individual circumstances. The removal of the bar more than 10 years after the initial procedure has been observed in certain instances, although such cases are relatively uncommon. The decision to remove the bar is usually based on factors such as the stability of the chest wall, the presence of any complications or discomfort associated with the bar, and the patient’s age. Our study contributes to the existing knowledge by specifically examining patients with a correction duration exceeding 3 years, providing valuable insights into the impact of delayed bar removal.

The removal of the Nuss bar is commonly performed as an outpatient procedure without complications. While the occurrence of massive bleeding during bar removal is rare, it presents a critical and life-threatening danger. Reports have documented instances of significant bleeding following bar removal, resulting from bleeding in the bar track, myocardial injury, lung laceration, and aortic laceration [22, 23]. While the incidence of complications related to bleeding during pectus bar removal is relatively low, it remains a significant concern [6, 22, 23]. The risk factors associated with major bleeding include migration of the bar, reoperation, erosion of the sternum, intrathoracic infection, or pericarditis. Considering patient safety and in the event of unforeseen major complications, it may be necessary to perform the pectus bar removal as an in-patient procedure, following a previously described protocol [16], especially in patients with risk factors for bleeding. In our study, no major bleeding was observed, and the overall complication rate was 4.8% (26/542). Among the rare instances of pneumothorax during Nuss bar removal, one patient in our cohort received intra-operative pleural drainage.

### Explanations of findings

Table 2 illustrates the analysis conducted after PSM, demonstrating the association of other surgical factors. We excluded patients due to lost follow-up, early removal of the bar related to intractable pain, wound infection, and allergy to the bar after the Nuss procedure. The results showed significant differences in the age of bar removal (*P* < 0.001), duration of correction (*P* < 0.001), callus formation (*P* = 0.029), operation time (*P* = 0.026), and blood loss (*P* = 0.017) between the two groups. Nonetheless, no substantial disparities were observed in relation to gender, BMI, HI, bar number, duration of hospital stay, complications, or radiographic improvement. Notably, a significant variation in operation time post-PSM suggests a possible association with callus formation. Our study reported no significant radiographic improvement or major complications post-procedure. Additionally, observed *P*-values for HI (CXR) before repair (*P* = 0.408), HI (CXR) after removal (*P* = 0.398), and improvement after repair (%) (*P*= 0.132) did not reach statistical significance. These findings suggest that delaying bar removal does not yield additional image-based improvements, indicated by similar HI values pre- and post-removal. In our previous study, we investigated bar rotation by measuring the slope angle of bars in patients undergoing a procedure, finding that cases with a slope angle > 30° showed reduced clinical improvement [17]. However, due to a limited sample size and specific exclusion criteria, we could not comprehensively analyze outcomes post-bar removal, hindering significant observations in this subgroup. Importantly, the incidence of complications did not exhibit any statistically significant differences between the groups, both pre- (*P* = 0.325) and post-PSM (*P* = 0.648).

### Strengths and limitations

While this study offers valuable insights, it is crucial to recognize its limitations. The relatively small number of cases and its retrospective nature may have introduced inherent biases and limitations in data collection. To overcome these limitations, we suggest conducting larger multicenter studies and performing meta-analyses across multiple centers. The reliance on medical records and the potential for incomplete follow-up data may have impacted the accuracy and completeness of the results.

### Implications and actions required

Future research directions can be postulated based on the study findings. Prospective studies involving larger multicenter cohorts would provide a more robust analysis of the risks and benefits of delayed bar removal in the Nuss procedure. Long-term follow-up studies focusing on patient-reported outcomes, quality of life, and cosmetic results would further contribute to our understanding of the optimal duration of bar retention. Investigating the impact of individual patient factors on the outcomes of delayed bar removal could also provide valuable insights for personalized treatment approaches.

## Conclusions

This study provides valuable insights into the impact of the delayed removal of the Nuss bar. The data suggest that while delayed removal can increase operation time, callus formation rates, and intraoperative blood loss, it does not significantly affect the rate of surgical complications or the degree of postoperative improvement.

## Data Availability

The datasets generated and/or analyzed during the current study are not publicly available due to the consideration of patient data confidentiality and subsequent research but are available from the corresponding author on reasonable request.

## Abbreviations

PE: Pectus excavatum
IRB: Institutional Review Board
BMI: Body mass index
HI: Haller index
PSM: Propensity score matching
CXR: Chest radiograph
CDC: Clavien–Dindo classification
